# Transcriptomic Analysis Reveals Glycolysis and Gluconeogenesis Pathway Activation Underlying Growth Enhancement by Duck-Blood Protein Hydrolysate in Flowerhorn Cichlid Fish

**DOI:** 10.3390/ijms26199563

**Published:** 2025-09-30

**Authors:** Pimpisut Manassila, Papungkorn Sangsawad, Surintorn Boonanuntanasarn, Jirawadee Kaewda, Pakpoom Boonchuen, Sirawich Limkul, Chatsirin Nakharuthai

**Affiliations:** 1 School of Animal Technology and Innovation, Institute of Agricultural Technology, Suranaree University of Technology, 111 University Avenue, Muang, Nakhon Ratchasima 30000, Thailand; m6500122@g.sut.ac.th (P.M.); papungkorn@sut.ac.th (P.S.); surinton@sut.ac.th (S.B.); m6500115@g.sut.ac.th (J.K.); 2 School of Biotechnology, Institute of Agricultural Technology, Suranaree University of Technology, 111 University Avenue, Muang, Nakhon Ratchasima 30000, Thailand; pakpoom.b@sut.ac.th (P.B.); sirawich.limkul@hotmail.com (S.L.)

**Keywords:** feed additive, animal by-product, duck blood protein hydrolysate, flowerhorn cichlid, Amphilophus, RNA-seq, home aquarium

## Abstract

Protein hydrolysates have potential as sustainable functional feed ingredients or additives for the aquaculture industry. This study examined the growth-promoting effects of duck-blood protein hydrolysate (DBPH, <10 kDa) on the flowerhorn cichlid (*Amphilophus citrinellus* × *Cichlasoma trimaculatum*). Fish with an average weight of 3.24 ± 0.22 g were randomly assigned to four dietary treatments: a negative control (basal diet) and basal diets supplemented with 0.5%, 1%, and 2% DBPH. After 90 days of the feeding trial, growth parameters indicated that supplementation with 1% and 2% DBPH enhanced growth. However, the muscle composition and skin coloration did not differ significantly. Transcriptome sequencing of the liver tissue yielded 39.83 GB of high-quality clean data. De novo transcriptome assembly identified 32,824 unigenes, of which 21,385 were successfully annotated based on public databases. Differential expression analysis identified 269 upregulated and 232 downregulated genes. To clarify the growth-promoting effects of DBPH, five glycolysis/gluconeogenesis-related genes (*tpi*, *gapdh*, *pck1*, *ldh*, and *adh*) were validated by liver qRT-PCR, and the results were consistent with those of the transcriptomic analysis. These findings provide new insights into the mechanisms by which DBPH supplementation could enhance growth, as evidenced by alterations in glycolysis and gluconeogenesis pathways, indicating potential as a novel feed additive in aquaculture.

## 1. Introduction

Ornamental fishkeeping has become one of the most popular hobbies in the world and attracts millions of enthusiasts as it offers both aesthetic and mental health benefits, as well as economic opportunities for entrepreneurs [1,2]. As a result, ornamental fish culture is an exponentially growing business. The total global value reached 5.4 billion USD in 2021, and the predicted annual growth rate is 8.5% from 2022 to 2030 [3]. More than 50% of ornamental fish suppliers are located on the continent of Asia, including Japan, Singapore, Malaysia, and Thailand [4]. The flowerhorn cichlid (*Amphilophus citrinellus* (Günther, 1864) × *Cichlasoma trimaculatum* (Günther, 1867)) is a hybrid species of ornamental freshwater fish and one of the most popular, attracting fish keepers due to its distinctive appearance [5,6]. The price of flowerhorn cichlids depends on the uniqueness of the type of fish, its size, color, attributes, rarity, individual consumer preferences, and consumer demand across different market regions [7,8]. Generally, a rounded and large nuchal hump, vivid colors, and well-balanced body are the most desirable traits for consumers and can increase the value of fish [9,10]. In Thailand, the flowerhorn cichlid is widely recognized in domestic and international markets. The attractive physical traits serve as indicators of quality and uniqueness and are more pronounced in larger fish than smaller individuals, whose features are not yet fully developed. As a result, body size is an important factor in determining price, as with other fish species [11].

Similarly to other ornamental fish species, the fingerling phase of culture is a critical period due to the transition from live feed to artificial feed. This stage is particularly important because live feeds pose challenges such as seasonal availability and a risk of pathogen contamination [12,13]. For flowerhorn cichlids, this growth phase marks the onset of the development of colorful body patterns and nuchal hump, which makes it important in terms of economic value. From a metabolic and physiological perspective, it is also a crucial period for the development of the immune and digestive systems and may have long-term effects on growth and health. However, artificial feed can occasionally pose challenges in terms of palatability and nutritional quality. Furthermore, the supplementation of feed additives to promote growth and metabolic responses, especially carbohydrate metabolism, is a significant challenge as commercial artificial diets typically contain a high proportion of carbohydrates to reduce production costs. To address this issue, researchers have explored novel feed additives, and one promising option is protein hydrolysates derived from animal byproducts. Protein hydrolysates exhibit several advantageous properties, including improved digestion and assimilation compared to intact proteins. Thus, protein hydrolysates can serve as both functional ingredients and as precursors for protein synthesis [14,15,16,17]. In addition, low-molecular-weight peptides and free amino acids in hydrolyzed protein can enhance feed palatability and potentially stimulate fish-feed intake [18].

Velasco et al. [19] reported that dietary supplementation with swine-blood protein hydrolysates enhanced muscle-protein synthesis and supported growth in European seabass (*Dicentrarchus labrax*). Although this parameter is not used to directly assess the aesthetic value of ornamental fish, optimal nutrition can enhance muscle-tissue composition, which indirectly reflects overall health, vitality, growth, and development. Therefore, it is reasonable to assert that there is a relationship between muscle-tissue composition, health, and aesthetic traits in ornamental fish. Our previous study [20] successfully converted duck-blood waste from the food industry into functional protein hydrolysates at a pilot scale and achieved higher hydrolysate yield while preserving bioactive properties (WMHVR, YAHVR, MPFKY, PDDPR, and NKVHF). The results highlighted potential applications in the functional feed industry that could contribute to sustainable practices through valorization of waste-derived proteins. Manassila et al. provided robust evidence that dietary supplementation with duck-blood protein hydrolysate (DBPH) enhances the overall health of flowerhorn cichlids by improving humoral immune responses, reducing oxidative stress, and bolstering resistance to *Streptococcus agalactiae* [21]. However, previous studies have mainly focused on the health-promoting effects of DBPH, and its potential to enhance somatic growth and the underlying gene expression related to growth and metabolism remain unexplored. In teleosts, the liver fulfills multiple essential functions, including regulating carbohydrate, protein, and lipid metabolism; detoxification; bile production; nutrient storage; hormone synthesis and regulation; and immune function. Alterations in the composition of a fish’s diet can significantly affect metabolic responses in the liver, particularly the content of protein, which is essential for the growth and health of fish [22]. To understand the underlying mechanisms, transcriptomic analysis is a powerful tool for determination of the biological processes and metabolic pathways that are influenced by dietary supplementation [17].

Protein hydrolysates have been extensively studied in food fish, particularly in high-value species and in the context of dietary protein replacement, but their application for ornamental fish nutrition remains largely unexplored. Therefore, this study examined the incorporation of DBPH as a feed additive to enhance the efficacy of commercial diets, which typically exhibit lower digestibility than live feeds. The aim was to improve growth during the sensitive transition from live to formulated feed. The initial size of fish used in this study (approximately 3 cm in length) was suitable for the use of fine-powdered commercial diets. We evaluated the effects of low-molecular-weight DBPH on growth performance, muscle-tissue composition, coloration, and liver transcriptome in flowerhorn cichlids during a 90-day feeding trial. Transcriptomic profiling using high-throughput RNA-seq was performed to elucidate the underlying mechanisms of growth promotion. This research supports the valorization of duck-blood waste by evaluating its potential as a novel feed additive in aquaculture.

## 2. Results

### 2.1. Growth Performance

After 90 days of feeding, fish fed diets supplemented with 1% and 2% DBPH showed significantly improved growth performance compared to the control group. The improvements included the final weight, final length, weight gain, feed-conversion ratio, average daily gain, and specific growth rate (Table 1).

### 2.2. Sequencing and Annotation of Unigenes

To elucidate the effect of DBPH on the liver transcriptome, the liver tissue from the 2% DBPH group was analyzed using RNA sequencing and compared to that of the negative control group. Following quality control, 39.83 GB of clean data were obtained from six libraries: three from the control group and three from the 2% DBPH group. The libraries containing Q30 bases exceeded 94.23% of all data. A summary of clean data statistics is presented in Table 2.

**Figure 1:**
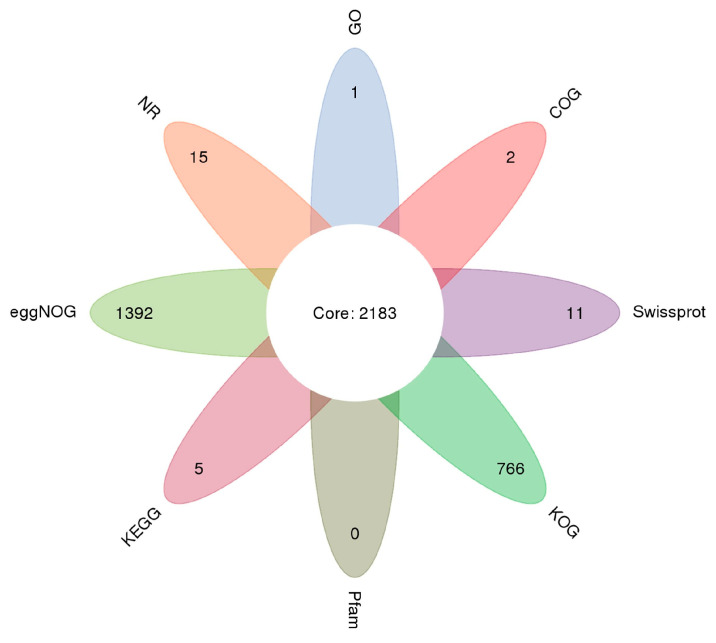
Petal diagram of unigene annotation.

After sequence assembly, 32,824 unigenes were generated, with an assembly yielding a unigene N50 value of 2151 bp (Table S1). As shown in Table S2, 21,385 unigenes were annotated using multiple databases, including the NR (20,719 unigenes), Swiss–Prot (9687), COG (3612), KOG (12,301), eggNOG (18,121), Pfam (14,272), Gene Ontology (GO) (18,093), and Kyoto Encyclopedia of Genes and Genomes (KEGG) (16,889) databases. The distribution of unigene annotations across different pathways is shown in a petal diagram of the unigene annotation (Figure 1).

### 2.3. Differentially Expressed Genes

**Figure 2:**
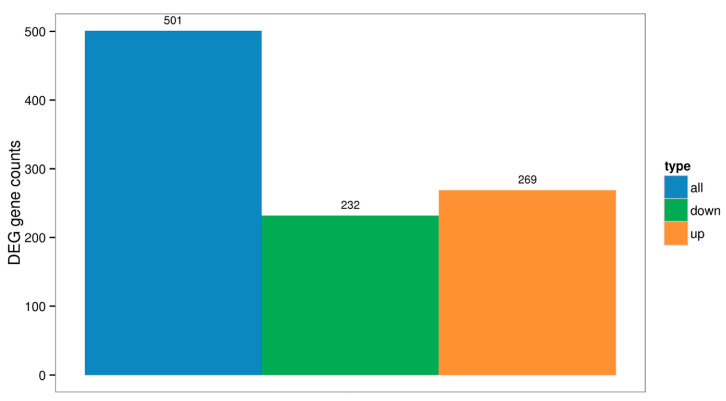
Histogram of differential gene statistics (Control vs. DBPH).

**Figure 3:**
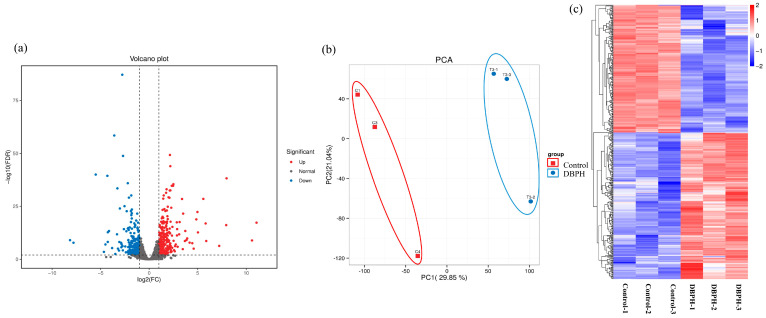
Volcano plot on differential expression (**a**) with dashed lines indicating log2 fold change and FDR cutoffs; Principal component analysis (PCA) for the correlation among samples (**b**); Hierarchical clustering of differentially expressed genes (**c**).

A total of 501 differentially expressed genes (DEGs) were identified in the liver between the control group and the group receiving 2% DBPH, of which 269 were upregulated, and 232 were downregulated. The number of differentially expressed genes in each category is illustrated in a histogram in Figure 2. A high number of unigenes with differential expression between these groups was observed, as shown in the volcano plot in Figure 3a.

Principal component analysis (PCA) (Figure 3b) revealed scatter points corresponding to three replicates in both the negative control and 2% DBPH groups that were clustered tightly within their respective groups. This indicated consistent expression profiles within groups and clear separation between them. Hierarchical clustering of DEGs was performed to examine expression patterns across samples, and the resulting heatmap (Figure 3c) confirmed the distinct sample clustering according to treatment groups with replicates grouping closely within each group.

### 2.4. GO Enrichment Analysis of DEGs

**Figure 4:**
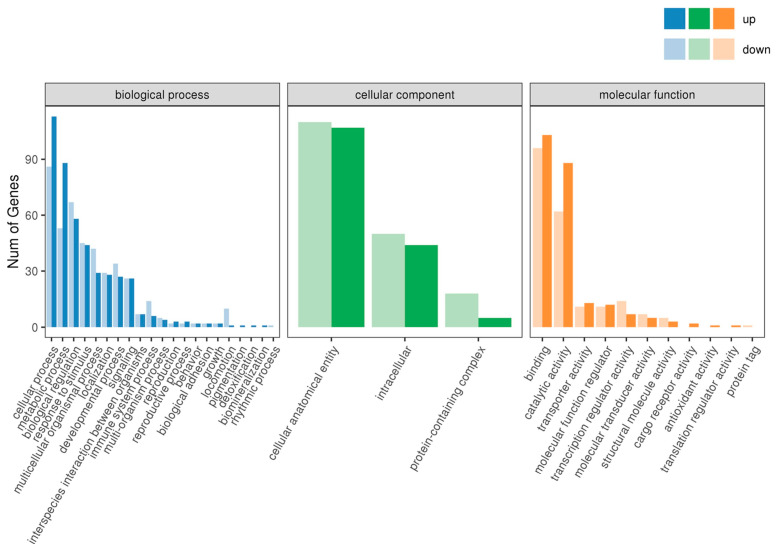
Statistics of GO annotation classification on differentially expressed genes.

We used the GO database to categorize the DEG functions into three main clusters: biological processes, cellular components, and molecular functions. All DEGs in the liver were distributed across 35 GO categories (Figure 4). These DEGs were annotated for 21 biological processes, and the most significant enrichments were observed in cellular processes, metabolic processes, biological regulation, and responses to stimuli. In total, 3 GO categories were found in cellular components: cell anatomical entities, intracellular entities, and protein-containing complexes. Regarding molecular functions, 11 GO categories were identified, of which binding and catalytic activity were the most enriched.

### 2.5. KEGG Enrichment Analysis of DEGs

**Figure 5:**
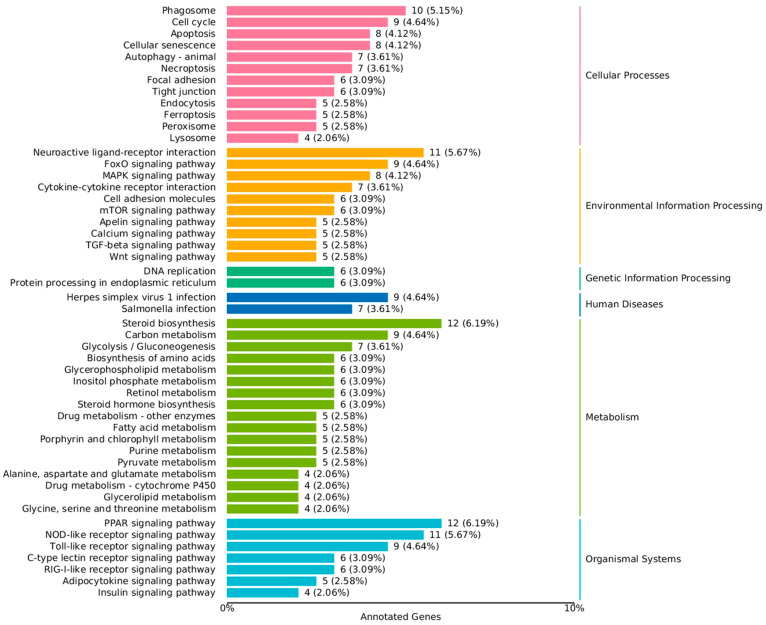
KEGG classification on DEGs; The left ordinate is the name of the KEGG metabolic pathway, the right ordinate represents the first-class classification name corresponding to the annotated pathway, and the abscissa is the number of genes annotated to this pathway and their proportion to the total number of annotated genes.

In the liver, KEGG pathway analysis categorized the data into six major categories: cellular processes (12 pathways), environmental information processing (10 pathways), genetic information processing (2 pathways), human diseases (2 pathways), metabolism (17 pathways), and organismal systems (7 pathways). Among these, the metabolic pathways had the highest number of DEGs, and the top three significantly enriched pathways in this category were steroid biosynthesis, carbon metabolism, and glycolysis/gluconeogenesis (Figure 5).

### 2.6. Validation of DEGs by qRT-PCR

**Figure 6:**
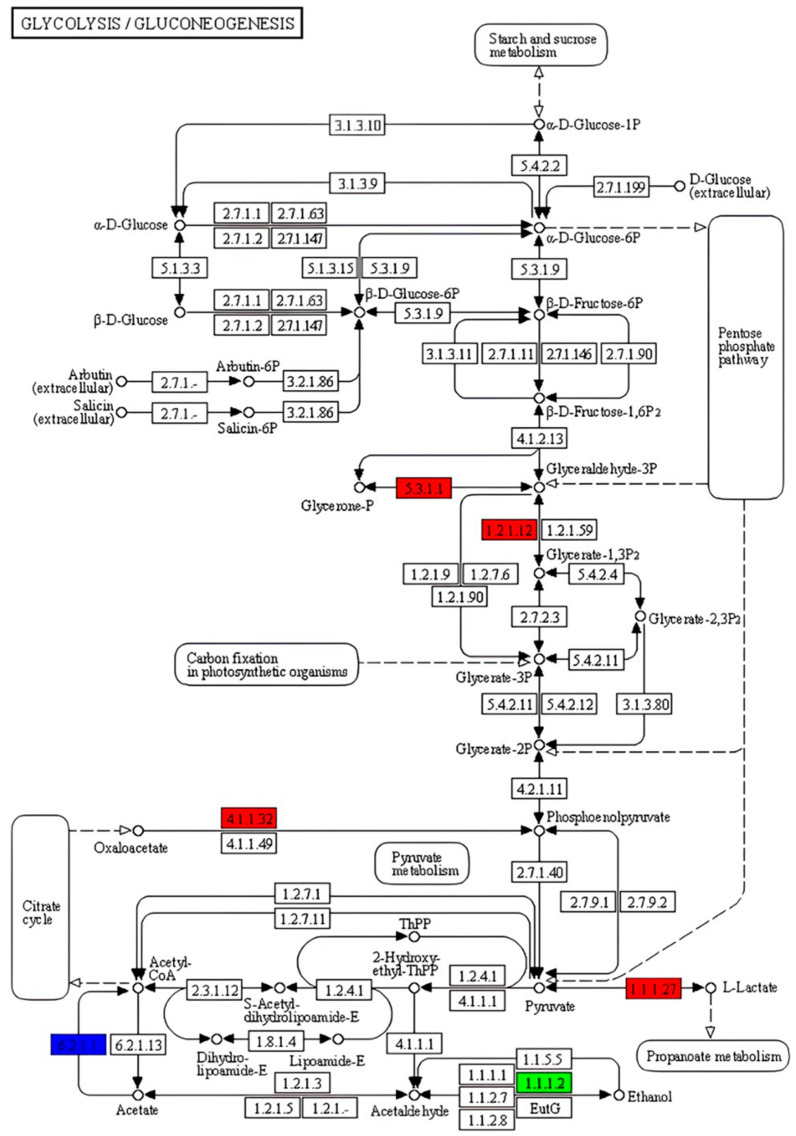
KEGG annotation analysis of the glycolysis/gluconeogenesis pathway. Note: Relative to the control group, the nodes colored in red represent the enzymes related to up-regulated genes and the green ones represent that down-regulated gene. Blue ones represent enzymes related to both up- and down-regulated genes. The number in the box stands for the EC number.

Glycolysis and gluconeogenesis are key pathways for energy availability and metabolic balance in fish. Thus, to gain insight into the effect of DBPH on growth performance, the DEGs involved in these pathways were identified in the broader metabolic network. qRT-PCR was performed to validate five genes–triose-phosphate isomerase (*tpi*), glyceraldehyde 3-phosphate dehydrogenase (*gapdh*), phosphoenolpyruvate carboxykinase 1 (*pck1*), lactate dehydrogenase (*ldh*), and alcohol dehydrogenase (*adh*)–that were up- or down-regulated in these pathways to confirm the transcriptomic result (Figure 6).

**Figure 7:**
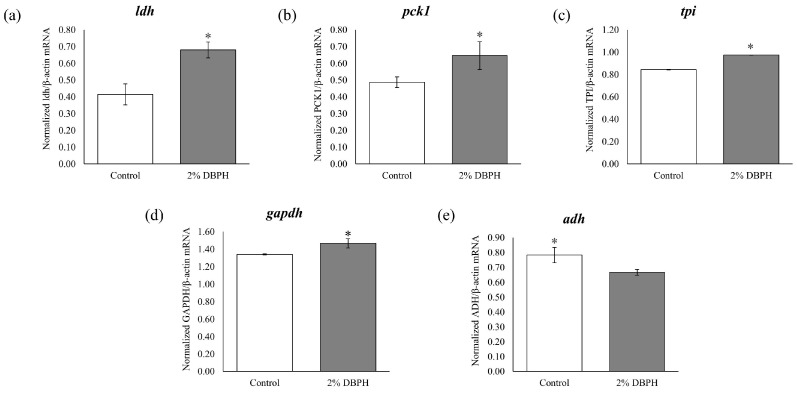
Quantitative real-time PCR analysis of *idh* (**a**); *pck1* (**b**); *tpi* (**c**); *gapdh* (**d**); and *adh* (**e**) expression in the liver of flowerhorn fish after the 90-day feeding trial. Bars marked with asterisks denote statistically significant differences between the control and 2% DBPH groups (*p* < 0.05).

The qRT-PCR expression profiles of the candidate genes from the liver of the experimental fish were consistent with the RNA–Seq results, as illustrated in Figure 7.

### 2.7. Proximate Analysis of Muscle and Skin Color Measurement

Proximate and color analyses were conducted to evaluate the effect of DBPH on muscle composition (protein, moisture, fat, and ash contents) and coloration. The findings indicated that no parameters differed significantly, as shown in Table 3.

## 3. Discussion

Animal byproducts have gained increasing interest among animal feed nutritionists as an alternative protein source to address the growing protein demand in the feed industry. Protein hydrolysates are valued for their unique composition and bioavailability and offer several benefits when used in animal feed. These benefits include enhanced digestibility that improves nutrient uptake and efficiency, especially for young or stressed animals with underdeveloped or compromised digestive systems [23,24,25].

Our previous study successfully addressed the valorization of duck-blood into bioactive protein hydrolysates, and the process was scaled up to the pilot production scale, which indicated industrial viability [20]. In addition, low-molecular-weight DBPH contains bioactive peptides that enhance immune function, which could potentially reduce the incidence of disease by increasing antioxidant activity and upregulating immune-related genes that are involved in resistance to pathogens [20,21]. Their high-quality amino-acid profile also promotes growth performance, muscle development, and overall growth, which is particularly advantageous in aquaculture [26]. Although studies on feed nutrition indicate that protein hydrolysates support livestock and aquaculture systems, higher proportions often reduce feed palatability [27].

We investigated the growth-promoting properties of low-molecular-weight DBPH (<10 kDa), and the results showed that incorporating 1% and 2% DBPH into diets positively influenced growth-performance parameters under experimental conditions. This effect may be attributed to the enhanced bioavailability, digestibility, and absorption of DBPH, which potentially provides a more readily available source of amino acids that improves nutrient absorption and promotes growth [28]. Additionally, since it is “pre-digested,” protein hydrolysate could reduce metabolic demands and allow more energy to be directed toward growth rather than digestion [29,30]. Moreover, lower inclusion levels (1% or 2%) could maintain feed palatability, thus maintaining palatability for taste-sensitive species while promoting growth. This finding is consistent with the results of Suma et al. [31], who demonstrated that incorporating 1% and 2% fish protein hydrolysate into diets enhanced palatability for Pabda (*Ompok pabda*) fingerlings.

DEGs were identified in the livers of experimental fish to elucidate the molecular mechanisms underlying growth improvement and pathways affected by DBPH. This study represents the first report of transcriptome data for flowerhorn cichlids. A total of 32,824 unigenes were assembled, of which 21,385 were successfully annotated. GO and KEGG analyses were employed to examine the total of 501 DEGs. Compared to steroid biosynthesis and carbon metabolism, glycolysis and gluconeogenesis are the most directly relevant pathways for supporting fish growth. These pathways regulate energy availability and provide intermediates for anabolic processes such as protein synthesis and muscle development. The increased supply of readily digestible amino acids from DBPH provides a significant energy source for flowerhorn cichlids that influences glycolysis and gluconeogenesis through modulation of nutrient utilization and metabolic flux. These results are consistent with previous studies indicating that dietary inputs strongly shape metabolic pathway activity in fish [22,32,33,34].

The five genes *tpi*, *gapdh*, *pck1*, *ldh*, and *adh* were selected based on a combination of their significant differential expression and their known biological relevance to glycolysis/gluconeogenesis pathways, which are among the pathways most strongly associated with growth and metabolism. The results showed that four of the genes (*tpi*, *gapdh*, *pck1*, and *ldh*) were upregulated, while *adh* was downregulated. In the livers of fish, an increase in the expression of *tpi*, *gapdh*, *pck1*, and *ldh* genes suggests metabolic adjustments in response to the dietary intervention [22,35,36]. The upregulation of *tpi* and *gapdh* suggests enhanced glycolytic flux, which supports ATP production and provides intermediates for anabolic pathways. Increased *ldh* expression facilitates the conversion of pyruvate to lactate and indicates a shift toward anaerobic glycolysis to sustain energy production under oxygen-limited conditions or heightened metabolic demand post-feeding [37]. In addition, *pck*1 is a crucial gluconeogenic enzyme that converts oxaloacetate to phosphoenolpyruvate.

The upregulation of *pck1* could imply the activation of gluconeogenesis and could potentially maintain the availability of glucose for tissues with continuous demand [38]. These transcriptional changes collectively imply a coordinated metabolic response that combines ATP balance, substrate availability, and nutrient-sensitive growth-signaling pathways. This process may involve hormones such as insulin and IGF-1 and ultimately supports tissue growth and biosynthesis processes. The downregulation of *adh* may be attributed to feeding DBPH, which is a protein-rich additive as opposed to an alcohol source, which reduced the need for ethanol metabolism [39]. Feeding low-molecular-weight DBPH may provide readily digestible peptides and promote an integrated metabolic response where glycolysis and gluconeogenesis are fine-tuned to balance energy requirements and nutrient assimilation.

In addition, the enrichment of genes associated with the steroid biosynthesis pathway suggests that DBPH may influence the endocrine regulation of somatic growth, which potentially occurs through the modulation of cholesterol-derived hormones that are involved in the GH–IGF axis [40]. Furthermore, the upregulation of genes related to carbon metabolism indicates enhanced central metabolic activity, which facilitates ATP production and the synthesis of amino acids and nucleotides that are required for tissue development [41]. Together with glycolysis and gluconeogenesis, these pathways suggest that DBPH may promote growth by enhancing enzyme activity, stimulating energy metabolism, and supporting regulatory mechanisms that are involved in tissue proliferation.

Muscle composition and skin coloration may reflect indirect effects of nutrition, as indicated by improvements in the overall health and well-being of the fish [19]. Parameters such as protein and fat content are closely associated with growth performance and offer insight into how effectively fish metabolize nutrients from their diet [42]. In addition, vivid coloration is an important factor that reflects the impact of feed ingredients and determines the market value or price of ornamental fish. No significant changes in skin color or muscle composition were detected, which indicates that DBPH did not directly influence these traits, and its primary effects may instead occur through alternative physiological or metabolic pathways by providing readily absorbable nutrients that help maintain normal physiological function. The knowledge gained from this study provides substantial evidence supporting DBPH as a novel growth-promoting feed additive that accelerates fish growth and the development of appearance traits, which could satisfy consumer preferences and potentially increase market value.

## 4. Materials and Methods

### 4.1. Experimental Design and Diet Preparation

Low-molecular-weight DBPH was prepared according to the method described by Manassila et al. [21]. All procedures involving flowerhorn cichlids were approved by the Ethics Committee of Suranaree University of Technology, Animal Care and Use Committee (approval no. SUT-IACUC-0010/2023). The experiments were performed using healthy flowerhorn cichlids with an initial weight of 3.24 ± 0.22 g and an initial length of 3.36 ± 0.13 cm, which represent the size at which the fish can begin feeding on fine-powdered commercial diets. Due to their aggressive behavior, each fish was housed individually in a 55-L rectangular glass aquarium throughout the experimental period.

During a 14-day acclimatization period, fish were fed a basal diet ad libitum twice daily at the University Farm of Suranaree University of Technology, Nakhon Ratchasima, Thailand. After the acclimatization, a completely randomized design was employed. The experiment included four treatment groups: a negative control (basal diet) and three groups receiving basal diets supplemented with 0.5%, 1%, or 2% DBPH, with each treatment having eight replicates (*n* = 8). Throughout the 90-day experiment, the fish were fed twice daily and kept in aquarium tanks with temperature control, aeration, and water filtration systems. The water quality parameters were monitored daily, including pH (7.0–7.6), dissolved oxygen (DO) (5.0–6.5 mg/L), and ammonia (NH_3_) (<0.05 mg/L).

### 4.2. Assessment of Growth Performance

At the end of the 90-day feeding trial, the growth performance parameters were measured and calculated, including final weight (FW), final length (FL), weight gain (WG), average daily growth (ADG), specific growth rate (SGR), and feed conversion ratio (FCR):

WG (g) = Final weight − Initial weight

SGR (% day^−1^) = [(log(Final weight) − log(Initial weight))/Number of days] × 100

ADG (g day^−1^) = [Final weight − Initial weight]/Days

FCR = Feed intake/Weight gain

### 4.3. Total RNA Extraction

After the feeding trial, the effect of the DBPH on the liver transcriptome was investigated by extracting total RNA from liver samples in three replicates (*n* = 3) from the control and 2% DBPH groups. The 2% supplementation level was selected because it corresponded to the level previously shown to support optimal health, although both 1% and 2% DBPH enhanced growth [21]. Total RNA was extracted using GENEzol reagent (Geneaid Biotech Ltd., New Taipei City, Taiwan) and a conventional phenol–chloroform extraction method. The quantity and quality of RNA were assessed using a NanoDrop 2000/2000c Spectrophotometer (Thermo Scientific, Waltham, MA, USA) and agarose gel electrophoresis to confirm that the RNA was intact, free from contaminants, and of sufficient quality for RNA sequencing.

### 4.4. Library Preparation

RNA samples were prepared using 1 µg of qualified RNA as the input material. In accordance with the manufacturer’s recommendations, libraries were prepared using the NEBNext Ultra RNA Library Prep Kit for Illumina (New England Biolabs, Ipswich, MA, USA), and index codes were incorporated to associate sequences with each sample. Briefly, mRNA was purified from total RNA using poly-T oligo-attached magnetic beads. Fragmentation was performed with divalent cations at an elevated temperature in NEBNext First Strand Synthesis Reaction Buffer (New England Biolabs, Ipswich, MA, USA). First-strand cDNA was synthesized with a random hexamer primer and M-MuLV reverse transcriptase, followed by second-strand synthesis using DNA polymerase I and RNase H. The remaining overhangs were converted to blunt ends through exonuclease and polymerase activities.

Following the adenylation of the 3′ ends of the DNA fragments, NEBNext Adaptors with a hairpin loop structure were ligated to facilitate hybridization. cDNA fragments preferentially measuring 240 bp in length were purified using the AMPure XP system (Beckman Coulter, Brea, CA, USA). Next, 3 µL of USER Enzyme (New England Biolabs, Ipswich, MA, USA) were applied to size-selected adaptor-ligated cDNA at 37 °C for 15 min, followed by 5 min at 95 °C prior to PCR.

PCR was conducted using Phusion High-Fidelity DNA polymerase, universal PCR primers, and Index (X) Primer. The PCR products were purified with the AMPure XP system, and library quality was evaluated using the Agilent Bioanalyzer 2100 system (Agilent Technologies, Santa Clara, CA, USA). Clustering of the index-coded samples was performed on a cBot Cluster Generation System using TruSeq PE Cluster Kit v3-cBot-HS (Illumina Inc., San Diego, CA, USA) according to the manufacturer’s instructions. The library preparations were then sequenced on an Illumina Novaseq X, and paired-end reads were generated.

### 4.5. Bioinformatics Analyses

Unigene sets for the samples were generated by assembling. Library quality was assessed according to insert distribution, randomness, and saturation analysis. High-quality libraries were then used for bioinformatic analyses, including unigene expression quantification, gene structure characterization, DEG analysis, functional annotation of DEGs, and functional enrichment analysis.

### 4.6. Identification of DEGs and Functional Annotation

DEGs were conducted using the DESeq R package (version 1.10.1). *p*-values were adjusted using the Benjamini and Hochberg methods to control the false discovery rate. Genes with an adjusted *p*-value less than 0.05 identified by DESeq were considered differentially expressed. The DEGs were analyzed using GO enrichment and KEGG pathway enrichment analysis tools.

### 4.7. Validation of DEGs by qRT–PCR

Based on the RNA sequencing data, genes associated with the glycolysis/gluconeogenesis pathways were selected to validate the effects of low-molecular-weight DBPH as a feed additive. The genes included *tpi*, *gapdh*, *pck1*, *ldh*, and *adh*. The validation was performed using qRT-PCR analysis.

First-strand cDNA synthesis was conducted using the Viva cDNA Synthesis Kit (Vivantis Technologies, Selangor, Malaysia) according to the manufacturer’s instructions. The primers used in this experiment were designed based on transcriptome data assembled with Trinity from RNA sequencing results, and *β*-actin was used as the internal reference for normalizing gene expression levels. Table S3 presents the primers used and the sequence data for qRT-PCR of each gene employed.

Standard plasmids were cloned to quantify gene expression levels. Briefly, each target gene was amplified under the following conditions: 95 °C for 5 min, 95 °C for 30 s, 55 °C for 30 s, 72 °C for 1.5 min for 35 cycles; and 95 °C for 5 min. The PCR products were purified using a FavorPrep GEL/PCR Purification Kit (Farvogen Biotech Corp, Ping Tung, Taiwan) according to the manufacturer’s instructions and cloned into the pGEM T-Easy plasmid (Promega Corporation, Madison, WI, USA). The positive colony PCR clones were extracted using the FavorPrep plasmid extraction mini kit (Farvogen Biotech Corp., Ping Tung, Taiwan) according to the manufacturer’s recommendations. *Eco*RI restriction enzyme (New England Biolabs, Ipswich, MA, USA) was used to validate the insert DNA and confirm the accuracy of the sequence for each target gene using sequencing methods provided by Macrogen (Seoul, Republic of Korea).

The synthesized cDNA was used as a template for qRT-PCR analysis. The analysis was performed in triplicate on a CFX Opus Real-Time PCR System (Bio-Rad Laboratories, Hercules, CA, USA) with THUNDERBIRD SYBR qPCR Master Mix (TOYOBO, Osaka, Japan) according to the manufacturer’s protocol. A melting-curve analysis was conducted at the end to verify the specificity of the amplification. The mRNA level was quantitatively analyzed as described by Nakharuthai et al. [43].

### 4.8. Proximate Analysis

All experimental diets (*n* = 3) and muscle tissue of flowerhorn cichlids from each treatment (*n* = 8) were analyzed for protein, moisture, fat, and ash contents according to the methods outlined by the Association of Official Analytical Chemists [44]. The results were expressed on a dry-weight percentage basis. Briefly, moisture content was determined by drying samples in an oven at 105 °C until a constant weight was achieved. The ash content was analyzed by incinerating the sample in a muffle furnace until a constant weight of ash remained. The crude fat content was analyzed using the chloroform–methanol extraction method. The crude protein content was analyzed using the Kjeldahl method with an automated Kjeltec 2200 nitrogen analyzer (FOSS, Hillerød, Denmark).

### 4.9. Assessment of Skin Coloration

The skin color of all live fish (*n* = 8) from each experimental group was detected at an exact location on the operculum, which displays the most intense coloration on their bodies. The detection was performed using a portable hunterlab color quest XE colorimeter (HunterLab, Reston, VA, USA). Three dimensions of color were determined: L* (lightness), which represents transparency and brightness, a* (red–green), which ranges from red to green, and b* (blue–yellow), which ranges from blue to yellow.

### 4.10. Statistical Analysis

Statistical analyses were conducted using SPSS version 25.0 (IBM Corp., Armonk, NY, USA). The results are presented as the mean ± standard error of the mean (SEM). A one-way analysis of variance (ANOVA) was performed using a completely randomized design (CRD) to evaluate differences between group means with Tukey’s multiple comparison test and a significance threshold of *p* < 0.05. An independent sample *t*-test was conducted to evaluate the differences between the negative control group and the 2% DBPH group for the qRT-PCR analysis (*p* < 0.05).

## 5. Conclusions

Low-molecular-weight DBPH could be used as a more accessible source of small peptides and amino acids. The increased availability of amino acids enhances nutrient absorption, supports growth, and influences metabolic processes by regulating the expression of genes associated with glycolysis and gluconeogenesis, thereby impacting growth performance of flowerhorn cichlids. Furthermore, the use of duck blood, a byproduct from slaughterhouses, could contribute to reducing industrial waste by maximizing its beneficial application, which could support a zero-waste approach. Nevertheless, future studies should incorporate longer feeding trials, histological assessments, enzymatic growth markers, greater biological replication, and metabolomic approaches. These additions could enhance the depth of the analyses and provide more comprehensive understanding of the mechanisms underlying the growth-promoting effects of DBPH, particularly under conditions that are relevant to field conditions.

## Figures and Tables

**Table 1:** Growth performance of flowerhorn fish fed experimental diets for 90 days.

Parameters	Diets			
	Control	0.5% DBPH	1% DBPH	2% DBPH
FW (g)	26.16 ± 0.53 ^a^	26.39 ± 0.47 ^a^	29.39 ± 0.24 ^b^	29.47 ± 0.32 ^b^
FL (cm)	8.38 ± 0.47 ^a^	9.70 ± 0.33 ^b^	10.93 ± 0.15 ^b^	10.87 ± 0.14 ^b^
WG (g)	22.92 ± 0.53 ^a^	23.15 ± 0.46 ^a^	26.15 ± 0.24 ^b^	26.23 ± 0.32 ^b^
FCR	3.03 ± 0.07 ^b^	2.97 ± 0.06 ^b^	2.74 ± 0.02 ^a^	2.67 ± 0.03 ^a^
ADG (g day^−1^)	0.25 ± 0.01 ^a^	0.26 ± 0.01 ^a^	0.29 ± 0.00 ^b^	0.29 ± 0.00 ^b^
SGR (% day^−1^)	0.25 ± 0.02 ^a^	0.26 ± 0.02 ^a^	0.29 ± 0.01 ^b^	0.29 ± 0.01 ^b^

Abbreviations: DBPH = Low molecular weight duck blood protein hydrolysate; FW = Final weight; FL = Final length; WG = Weight gain; FCR = Feed conversion ratio; ADG = Average daily gain; SGR = Specific growth rate. Data are presented as mean ± SEM (*n* = 8). Means with different superscripts in each row differ significantly (*p* < 0.05).

**Table 2:** Clean data statistics.

Treatment	Clean Reads	Clean Base	GC Content (%)	% ≥Q30
Control1	20,334,724	6,092,042,946	49.72	94.40
Control2	21,970,953	6,582,712,088	49.90	94.43
Control3	22,642,113	6,783,876,573	50.04	94.23
2%DBPH1	21,972,991	6,583,108,021	49.64	94.65
2%DBPH2	22,239,091	6,662,959,823	49.76	94.67
2%DBPH3	23,773,994	7,121,481,778	49.56	94.86

**Table 3:** Colorimetric parameters and proximate composition of muscle.

Parameters	Diets			
	Control	0.5% DBPH	1% DBPH	2% DBPH
L*	37.96 ± 2.43	36.14 ± 2.07	41.67 ± 1.60	42.29 ± 1.01
a*	9.65 ± 1.01	9.79 ± 1.78	10.43 ± 0.18	11.73 ± 1.00
b*	10.37 ± 2.58	10.74 ± 3.41	9.99 ± 0.37	9.58 ± 2.05
Crude protein (%)	43.65 ± 1.26	45.22 ± 0.47	47.23 ± 1.30	47.64 ± 1.02
Crude lipid (%)	7.22 ± 0.52	5.20 ± 0.50	6.66 ± 0.52	5.90 ± 0.32
Moisture (%)	71.34 ± 2.30	72.34 ± 2.57	71.16 ± 2.30	72.54 ± 1.34
Ash (%)	13.08 ± 1.07	17.04 ± 1.29	14.29 ± 0.33	18.87 ± 1.15
Other compounds (%)	10.33 ± 0.50	8.49 ± 0.45	9.18 ± 0.44	7.58 ± 0.82

Abbreviations: DBPH = Low-molecular-weight duck blood protein hydrolysate; L* = lightness; a* = redness; b* = yellowness. Data are presented as mean ± SEM. No significant differences were observed (*p* ≥ 0.05).

## Data Availability

The data are available from the corresponding author upon reasonable request. The RNA sequencing data are available in SRA database, accession PRJNA1281550.

## Supplementary Materials

The supporting information can be downloaded at: https://www.mdpi.com/article/10.3390/ijms26199563/s1.
