# Supplementary material for: Transcriptomic Analysis Reveals Glycolysis and Gluconeogenesis Pathway Activation Underlying Growth Enhancement by Duck-Blood Protein Hydrolysate in Flowerhorn Cichlid Fish
Source: Int J Mol Sci. 2025 Sep 30;26(19):9563. doi: 10.3390/ijms26199563 (PMC12525348; doi:10.3390/ijms26199563)
Supplement: Supplementary file 1 [file ijms-26-09563-s001.zip › ijms-3828384-supplementary.pdf]

Table S1. List of Primers Used and Target Gene Sequences for qRT-PCR Analysis

**Name**	**Primer 5'-3'**	**Sequence data**	**Size (bp)**
*tpi*F<br>*tpi*R	GTGAGGGAGTGGCTGAAGAC<br>AAACTCTGGCTTCAGGGAGC	**GTGAGGGAGTGGCTGAAGAC**CAATGTATCCGAGCCTGTAGCCAACTCTGTAAGG<br>ATCATCTACGGAGGTTCTGTGAGAGGTGATAGCTGCAGAGAACTTGCCACCCAGAA<br>GGATATTGATGGTTTCATTGTGGGAAAAG**GCTCCCTGAAGCCAGAGTTT**	159
*gapdh*F<br>*gapdh*R	CTTCCTGCACAACCAACTGC<br>CTTGCCATTGAGCTCTGGGA	**CTTCCTGCACAACCAACTGC**CTGGCACCCCTGGCCAAGGTCATCCATGACAACTT<br>TGGCATCATTGAGGGCCTGATGAGCACAGTTCACGCCATCACTGCCACTCAGAAGA<br>CCGTGGACGGTCCCTCTGGTAAGCTGTGGAGGGACGGCCGTGGTGCCAGCCAGAAC<br>ATCATCCCCGCTTCTACTGGCGCTGCCAAAGCTGTCGGCAAGGTCA**TCCCAGAGCT<br>CAATGGCAAG**	233
*pck*1F<br>*pck*1R	ATGGCTTTTTCGGTGTTGCC<br>TTGGGATGAGCACAGGGTTC<br><br><br><br>TTGGGATGAGCACAGGGTTC	**ATGGCTTTTTCGGTGTTGCC**CCCGGCACCTCAGCCAAAACCAACCCAAACGCAAT<br>GGCAACCATCATCAAGAACACCATCTTCACAAACGTTGCGGAGACGAGCGACGGG<br>GGCGTGTACTGGGAGGGAATGGACGAGTCACTGCCTGAGGGAGTCACCATCACAT<br>CCTGGAAGAACAAACCGTGGAGCTCAGATGATGGC**GAACCCTGTGCTCATCCCA<br>A**	220
*ldh*F<br><br><br><br><br>*ldh*R	AAGCTCCACCTGCATCCTTC<br><br><br><br><br>AATGGCCCAGGAAGTGTAGC	**AAGCTCCACCTGCATCCTTC**TAGCTGCCATGGCTGGATCATCGGAGAGCATGGAG<br>ACTCCAGCGTACCTGTGTGGAGTGGTGTGAATGTTGCTGGAGTTTCTCTTCAAGGCC<br>TCAACCCAAACATGGGGACTGATGGTGACCAAGAGAACTGGAAGGAAGTGCACAA<br>GATGGTGGTTGATGGAGCCTATGAGGTCATCAAGCTGAAGG**GCTACACTTCCTGG<br>GCCATT**	228
*adh*F<br><br><br><br>*adh*R	ACGAAGCCTATCCAGGAGG<br><br><br><br>CCCTTCAATGTTCGTCCCA	**ACGAAGCCTATCCAGG**AGGTTGTGGTGGAGATGACGGATGGAGGTGTGGACTAC<br>GCTCTGGAGTGTGTTGGAAGTCCAGCTATCATGAGTGCTGCACTTGAGTCCACAAG<br>AGATGCCTGGGGTACTTGTGTCATTGCTGGTTGGACAGAGACAGAAGCAATGAGCG<br>TTCCAGTCGAAAAGCTTCTGA**TGGGACGAACATTGAAGGG**	206
*β-actin*F<br><br><br>*β-actin*R	ACAGGATGCAGAAGGAGATCACAG<br><br><br>GTACTCCTGCTTGCTGATCCACAT	**ACAGGATGCAGAAGGAGATCACAG**CCCTGGCCCCATCCACCATGAAGATCAAGA<br>TCATTGCCCCACCTGAGCGTAAATACTCCGTCTGGATCGGAGGCTCCATCCTGGCCT<br>CCCTGTCCACCTTCCAGCAG**ATGTGGATCAGCAAGCAGGAGTAC**	155

Abbreviation; *tpi* = triose-phosphate isomerase, *gapdh* = glyceraldehyde 3-phosphate dehydrogenase, *pck*1 = phosphoenolpyruvate carboxykinase 1, *ldha* = lactate dehydrogenase A, and *adh* = alcohol dehydrogenase

Note: Bases in bold indicate the position and sequence of the primers.

Table S2. Statistics of assembly

Length Range	Transcript	Unigene
200-300	7,552(17.96%)	7,001(21.33%)
300-500	7,287(17.33%)	6,179(18.82%)
500-1000	8,836(21.01%)	6,992(21.30%)
1000-2000	8,829(20.99%)	6,349(19.34%)
2000+	9,556(22.72%)	6,303(19.20%)
Total Number	42,060	32,824
Total Length	56,461,701	39,297,697
N50 Length	2,354	2,151
Mean Length	1342.41	1197.22

Table S3 Summary of unigene annotation

Annotated Database	Annotated Number	300<=length<1000	length>=1000
COG_Annotation	3612	628	287800%
GO_Annotation	18093	5605	9918
KEGG_Annotation	16889	5084	9511
KOG_Annotation	12301	3258	7828
Pfam_Annotation	14272	3681	9562
Swissprot_Annotation	9687	2522	6334
TrEMBL_Annotation	21046	6930	10924
eggNOG_Annotation	18121	5743	9784
nr_Annotation	20719	6761	10839
All_Annotated	21385	7105	10981
